# Faster Diagnosis of Suspected Lower Respiratory Tract Infections: Single-Center Evidence from BIOFIRE FilmArray^®^ Pneumonia Panel Results vs. Conventional Culture Method

**DOI:** 10.3390/diagnostics16020342

**Published:** 2026-01-21

**Authors:** Beatrice Silvia Orena, Lisa Cariani, Elena Tomassini, Filippo Girardi, Monica D’Accico, Alessia Pirrone, Caterina Biassoni, Daniela Girelli, Antonio Teri, Marco Tonelli, Claudia Alteri, Annapaola Callegaro

**Affiliations:** 1 Clinical Microbiology and Virology Unit, Fondazione IRCCS Ca’ Granda Ospedale Maggiore Policlinico, 20122 Milan, Italy; beatrice.orena@policlinico.mi.it (B.S.O.); lisa.cariani@policlinico.mi.it (L.C.); ele6895@gmail.com (E.T.); filippo.girardi.21@gmail.com (F.G.); monica.daccico@policlinico.mi.it (M.D.); alessia.pirrone@policlinico.mi.it (A.P.); caterina.biassoni@policlinico.mi.it (C.B.); daniela.girelli@policlinico.mi.it (D.G.); antonio.teri@policlinico.mi.it (A.T.); marco.tonelli@policlinico.mi.it (M.T.); claudia.alteri@unimi.it (C.A.); 2 Department of Oncology and Hemato-Oncology, University of Milan, 20122 Milan, Italy

**Keywords:** respiratory infections, syndromic panel, conventional culture, resistance markers

## Abstract

**Background/Objectives**: Syndromic multiplex PCR assays such as BIOFIRE FilmArray^®^ Pneumonia (PN) panel enable rapid and simultaneous detection of bacterial and viral pathogens in respiratory specimens, improving diagnostic accuracy and patient management in lower respiratory tract infections (LRTIs). **Methods**: In this retrospective observational study, PN panel results in 410 bronchoalveolar lavage (BAL) samples from hospitalized patients with suspected pneumonia were analyzed and compared with those obtained using the conventional culture (CC) method. **Results**: The PN panel showed an overall positivity rate of 54%, detecting bacteria in 39.0% of samples, viruses in 7.1%, and atypical bacteria in 2.2%. Using the conventional culture (CC) method, 33.9% of samples tested positive. Overall, 83 (20.2%) samples that were positive by the PN panel were negative by CC, whereas only 14 specimens (3.4%) were positive by CC and negative by PN panel. The most frequently detected pathogen by both the PN panel and CC was *Staphylococcus aureus* (n = 67, 16.34% for PN; n = 40, 9.76% for CC). Regarding diagnostic performance, the PN panel demonstrated a sensitivity of 89.02%, a specificity of 97.86%, and an overall accuracy of 97.63%. Lower sensitivity values were observed only for the *Enterobacter cloacae* complex (57.14%) and the *Klebsiella pneumoniae* group (75%). Specificity exceeded 92% for all bacterial targets. **Conclusions**: The PN panel confirms enhanced pathogen detection and a shortened time-to-result. It serves as a valuable adjunct for the timely diagnosis of LRTIs, supporting antimicrobial stewardship through more precise and appropriate antibiotic selection.

## 1. Introduction

Lower respiratory tract infections (LRTIs) represent a significant public health threat and remain the world’s most deadly infectious disease after COVID-19. In 2021, LRTIs ranked as the fifth leading cause of death globally, accounting for 2.5 million deaths [1,2,3]. The etiology of LRTIs can be bacterial, viral, or fungal, depending on patient exposure and specific clinical risk factors. Moreover, the emergence and spread of multidrug-resistant (MDR) bacteria, including *Staphylococcus aureus*, *Pseudomonas aeruginosa, Klebsiella pneumoniae,* and other members of *Enterobacterales*, increase the complexity of treatment, leading to poor clinical outcomes, longer hospital stays, and higher mortality rates [4,5,6].

Currently, conventional culture (CC) is the reference method for isolation, identification, and antimicrobial susceptibility testing (AST) of respiratory bacteria [7]. Diagnostic performance of the CC method is variable due to antibiotic exposure before specimen collection, fastidious growth characteristics of specific bacteria, or overgrowth of resident microbiota, leading to reduced sensitivity and prolonged turnaround time (TAT) [8,9].

Syndromic testing has introduced the field of clinical microbiology to a new frontier of diagnostic capabilities, since it provides a highly powerful, fast, and accurate tool capable of simultaneously detecting a wide range of pathogens associated with the same clinical syndrome [7,10]. The BIOFIRE FilmArray^®^ Pneumonia (PN) panel (BioFire Diagnostics, LLC, Salt Lake City, UT, USA) is a multiplex real-time PCR assay designed to detect multiple bacterial and viral pathogens, as well as specific resistance genes from untreated sputum-like (including endotracheal aspirates) and bronchoalveolar lavage (BAL)-like samples. This assay delivers results in approximately 75 min, with only 5 min of hands-on time [11]. Several studies, evaluating the PN panel’s performance, highlighted an increase in positivity rate of 30–60% compared with the CC method [4,12,13,14]. Thus, a prompt and rapid laboratory diagnosis of LRTIs etiology and the definition of targeted antibiotic therapy is necessary to guide correct patient management [15,16].

In this study, we analysed PN panel results in hospitalized patients with suspected pneumonia and compared them with those obtained using the conventional culture (CC) method.

## 2. Materials and Methods

### 2.1. Study Population and Specimen Collection

This retrospective observational study was conducted at IRCCS Fondazione Ca’ Granda Ospedale Policlinico of Milan, between 1 January 2022 and 31 December 2024. A total of 410 respiratory specimens were collected from 343 patients > 18 years old (median age 60 years; 233 males) hospitalized in selected high-risk wards, including hematology, intensive care, medicine, and surgery. For patients with a prolonged hospital stay, each BAL repeated at one-week intervals was treated as an independent sample. The inclusion criteria were a clinical suspicion of pneumonia, performance of a BAL procedure, and availability of both the PN panel and the CC method conducted on the same BAL sample. Suspected pneumonia was defined as a clinical condition presenting with signs and symptoms consistent with pneumonia (e.g., fever, cough, sputum production, dyspnea, and abnormal breath sounds) in the absence of microbiological or radiological confirmation. Clinical information and definitive microbiological or radiological confirmation of pneumonia were not available at the time of sample inclusion.

Exclusion criteria included BAL performed for reasons other than suspected pneumonia, such as in lung donors or recipients, as well as cases in which the PN panel and CC method were performed on different BAL samples. All BAL samples were collected via bronchoscopy following standard clinical protocols and transported to the microbiology laboratory within four hours of collection to preserve sample integrity.

Molecular testing using the BIOFIRE FilmArray^®^ Pneumonia (PN) Panel assay and conventional culture (CC) method was performed on the same BAL sample.

### 2.2. BIOFIRE FilmArray^®^ Pneumonia Panel

The PN panel is a multiplex polymerase chain reaction (PCR) system designed for the rapid detection of multiple bacterial and viral pathogens, as well as key antibiotic resistance genes, from a single specimen. This fully automated test identifies nucleic acids from a broad range of pathogens using a specialized test pouch that analyzes a respiratory sample in approximately one hour. Specifically, PN panel detects 15 common bacterial pathogens (*Acinetobacter calcoaceticus-baumannii* complex, *Enterobacter cloacae complex*, *Escherichia coli*, *Klebsiella pneumoniae* group, *Klebsiella aerogenes*, *Klebsiella oxytoca*, *Moraxella catarrhalis*, *Proteus* spp., *Serratia marcescens*, *Haemophilus influenzae*, *Pseudomonas aeruginosa*, *Staphylococcus aureus*, *Streptococcus pneumoniae*, *S. pyogenes*, and *S. agalactiae*), 3 atypical bacteria (*Chlamydia pneumoniae*, *Legionella pneumophila*, *Mycoplasma pneumoniae*), 8 respiratory viruses (influenza A and B, parainfluenza virus, respiratory syncytial virus, rhinovirus/enterovirus, human metapneumovirus, and coronavirus) and 7 genetic resistance determinants (blaCTX-M, blaIMP, blaKPC, blaNDM, blaOXA-48-like, blaVIM, mecA/C and MREJ). Results of common bacterial pathogens are reported semi-quantitatively as 10^4^, 10^5^, 10^6^, and >10^7^ genomic copies/mL, while atypical bacteria, viruses, and genetic resistance determinants are reported qualitatively as detected or not detected. Genetic resistance determinants are detected only when at least an applicable bacterium (i.e., a potential carrier of genetic resistance determinants) is also detected in the sample.

All PN panels were performed according to the guidelines provided by the manufacturer [11].

### 2.3. Conventional Culture (CC) Method

Microbiological diagnostics were performed in the respective routine microbiological laboratory following the current national standards and requirements.

All BALs that underwent PN panel testing were subsequently analysed using conventional culture (CC) method. No assessment of specimen quality was performed.

Microbiological cultures were performed by inoculating 90 µL of BAL on different growth media, including Columbia Agar with 5% sheep blood (COS), the selective media Columbia colistin-nalidixic acid (CNA) agar with 5% sheep blood and chocolate Haemophilus agar (HAE2), the selective and differential media MacConkey Agar (MCK) and Sabouraud Gentamicin Chloramphenicol 2 agar (SGC2) (bioMérieux, Marcy-l’Étoile, France). Plates were incubated in standard atmospheric and 5% CO_2_ enriched environment at 35 °C for 48 h and examined daily for bacterial growth. The grown bacteria were identified by matrix-assisted laser ionization/desorption time-of-flight mass spectrometry (VI-TEK^®^ MS PRIME MALDI-TOF, bioMérieux, France) [17]. *S. pneumoniae* was identified using the optochin disc diffusion test. A 5 µg optochin disc was placed on a CNA agar plate previously inoculated with 90 µL of BAL and incubated at 35 °C in 5% CO_2_ enriched environment for 18 h. An inhibition zone ≥ 14 mm was interpreted as sensitive and presumptive for *S. pneumoniae.* Antimicrobial susceptibility tests (ASTs) were performed using the disk diffusion method (Kirby-Bauer) for *Haemophilus influenzae* and *Moraxella catarrhalis* and the automated instrument Vitek-2 system (bioMérieux, France) [18] for all other bacterial isolate types. Results were interpreted using established EUCAST clinical breakpoints. *S. aureus* isolates were considered methicillin-resistant when cefoxitin MIC (Minimum Inhibitory Concentration) values > 4 mg/L and oxacillin MIC values > 2 mg/L. *Enterobacterales* were classified as CTX-M producers when cefotaxime MIC > 2 mg/L. For carbapenemase screening, a meropenem MIC cut-off of >0.125 mg/L was used [19].

### 2.4. Additional Tests for Resistance Markers on Isolated Bacterial Strains

The presence of resistance markers on isolated bacterial strains was confirmed by lateral flow immunoassay NG-Test^®^ and Eazyplex^®^ MRSA Plus assay.

The production of CTX-M and carbapenemases was determined using the qualitative lateral flow immunoassay NG-Test^®^ CTX-M Multi and NG-Test^®^ Carba-5 (NG Biotech, Guipry-Messac, France), respectively [20,21]. NG-Test^®^ CTX-M Multi was able to detect enzymes belonging to the five major CTX-M Groups (1, 2, 8, 9, and 25), including a total of 26 variants; while NG-Test^®^ Carba-5 was able to detect the five most common carbapenemase families (KPC, OXA-48, VIM, IMP, and NDM), including a total of 76 variants.

The eazyplex^®^ MRSAplus (Arrow Diagnostics, Seegene, Seoul, Republic of Korea), a manual in vitro diagnostic test based on loop-mediated isothermal amplification (LAMP) technology, was used for the qualitative detection of specific nucleic acid sequences of *Staphylococcus aureus*, methicillin resistance genes (*mecA* and *mecC*), and the Panton-Valentine leukocidin (PVL) gene from bacterial isolates, providing results in approximately 15–20 min.

### 2.5. Statistical Analysis

Demographic data, including age and sex, and hospital wards were collected retrospectively from medical records.

The conventional culture (CC) method was used as the standard-of-care (SOC) test for comparison with the PN panel. Positivity rates for each PN panel target were calculated as the proportion of samples testing positive for that target relative to the total number of tests performed. Culture positivity was calculated as the proportion of positive cultures among all tests. Concordance between PN panel and CC results was evaluated qualitatively based on the presence or absence of each pathogen. Molecular detection of resistance genes was compared with qualitative lateral-flow immunoassays to assess genotypic–phenotypic agreement. Sensitivity, specificity, positive predictive value (PPV), negative predictive value (NPV), accuracy, and their respective confidence intervals were calculated using the MedCalc Diagnostic Test Evaluation Calculator (MedCalc Software Ltd., Ostend, Belgium, Version 23.2.8; accessed 18 July 2025). Statistical analyses—including the Chi-square test, Shapiro–Wilk test, and Wilcoxon test—were performed in R (version 4.4.2). A *p*-value < 0.05 was considered statistically significant. All reported *p*-values are two-sided.

### 2.6. Ethics Statement

All patient data were anonymized, ensuring that specimens and isolates could not be linked to individual patients.

Because specimens used in this study were part of routine patient management without any additional sampling, this study did not need to be examined by an ethics committee, and patients’ informed consent was not required.

## 3. Results

### 3.1. Patient and Specimen Characteristics

During a 3-year period (2022–2024), a total of 343 patients hospitalized with suspected pneumonia were included; 238 (69.4%) were male. The median age was 60 years ([IQR]: 48–68), with no significant difference among the study years (*p* = 0.25). The distribution of males was also comparable across 2022, 2023, and 2024 (71.3%, 72.7%, and 65.5%, respectively; *p* = 0.42). In total, 410 specimens were analyzed by both the PN panel and CC method, with a slight yearly increase from 115 in 2022 to 133 in 2023 and 162 in 2024. The majority of samples were obtained from intensive care units (ICU) (245, 59.8%), followed by medicine wards (115, 28.0%), hematology units (31, 7.6%), and surgical wards (19, 4.6%). Although the distribution of specimens among the different hospital wards varied across the years, the difference did not reach statistical significance (*p* = 0.06) (Table 1).

### 3.2. Pathogens Detected with PN Panel and CC Method

**Figure 1:**
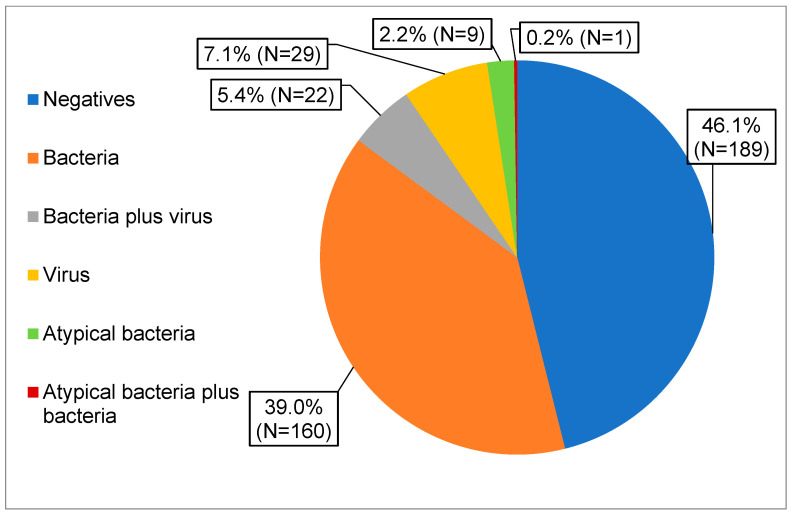
Distribution of all PN panel test results. Positive PN panel results were grouped by pathogen category, as described in the legend.

Of the 410 collected specimens, 221 (54%) tested positive for at least one respiratory pathogen included in the PN panel. This assay revealed that bacteria were the predominant pathogens, identified in 39.0% of samples (n = 160). Viruses were detected in 7.1% (n = 29) of samples and atypical bacteria in 2.2% (n = 9). Mono and polymicrobial infections were 64.3% (n = 142) and 35.7% (n = 79), respectively. The concurrent detection of bacterial and viral pathogens was observed in 22 samples (5.4%), while only one sample (0.2%) showed the mixed detection of bacterial and atypical bacterial pathogens (Figure 1).

**Figure 2:**
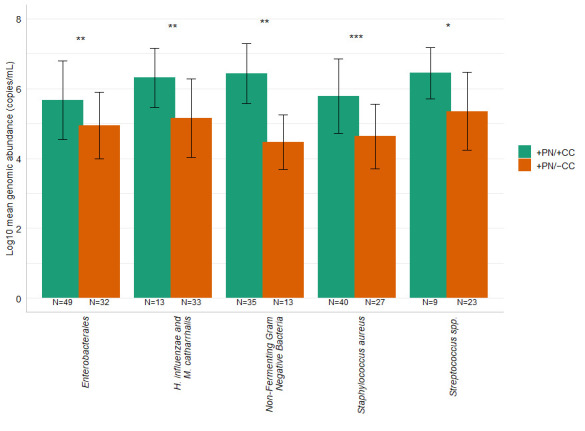
Comparison of mean genomic abundance detected by PN panel for +PN/+CC and +PN/−CC samples across bacterial groups. The chart displays the mean genomic abundance with error bars representing standard deviation, as well as the sample size (N) for each category. Asterisks highlight the statistical significance levels (*, *p* ≤ 0.05; **, *p* ≤ 0.01; ***, *p* ≤ 0.001). The *p*-values were calculated using the Wilcoxon test.

The most common detected pathogens were *S. aureus* (n = 67, 16.34%), *H. influenzae* (n = 42, 10.24%), *P. aeruginosa* (n = 39, 9.51%), *E. coli* (n = 28, 6.83%), *K. pneumoniae* group (n = 20, 4.88%) and *S. pneumoniae* (n = 25, 6.10%) (Table 2). *L. pneumophila* (n = 5, 1.22%) and *M. pneumoniae* (n = 4, 0.98%) were the most frequent detected atypical bacteria, while *Influenza Virus A* (n = 21, 5.12%), *Coronavirus* (229E, OC43, HKU1, NL63) (n = 12, 2.93%) and *Metapneumovirus* (n = 8, 1.95%) were the most frequent detected viral targets (Figure 2).

Using the CC method, 138 (33.9%) samples tested positive. Of these, 75.4% (n = 104) were monomicrobial infections. The most frequent bacteria were *S. aureus* (n = 40, 9.76%), *P. aeruginosa* (n = 35, 8.54%), *K. pneumoniae* group (n = 20, 4.88%), and *E. coli* (n = 19, 4.63%). Only 14 respiratory specimens (3.4%) tested positive for pathogens not included in PN panel targets. Of these, 43% (n = 6) were monomicrobial infections (including one sample that tested positive for filamentous fungi), while the remaining specimens showed the detection of more than one bacterium (n = 8). *Stenotrophomonas maltophilia* emerged as the predominant pathogen of this group (n = 8) (Table 3).

### 3.3. Comparison of Bacteria Detected by PN Panel Versus CC Method

Overall, regarding PN panel diagnostic performance, our results indicate a sensitivity of 89.02%, a specificity of 97.86%, and an accuracy of 97.63%. Sensitivity values were 100% for several species, including *H. influenzae*, *K. aerogenes*, *K. oxytoca*, *M. catarrhalis*, *Proteus* spp., *S. marcescens*, *S. aureus*, and *S. pneumoniae*. In contrast, lower sensitivity values were observed for the *E. cloacae* complex (57.14%) and the *K. pneumoniae* group (75%). Specificity values exceeded 92% for all bacterial targets, reaching 99.75% for *Proteus spp.* Similarly, accuracy remained above 92% for all bacterial species (Table 4).

The overall PN panel Positive Predictive Value (PPV) and Negative Predictive Value (NPV) of bacterial pathogen detections were 53.28% (146/274) and 99.69% (5858/5876), respectively. For *Acinetobacter calcoaceticus-baumannii* complex, *E. coli, K. pneumoniae* group, *Proteus* spp., *P. aeruginosa,* and *S. aureus,* the PPVs range from 59.70% to 83.30%. The PPVs for *H. influenzae* and *S. pneumoniae* were 26.20% and 36.00%, respectively. In line with that, 73.80% (31/42) of *H. influenzae* and 64.00% (16/25) of *S. pneumoniae* were identified exclusively by the PN panel. By contrast, the negative predictive values (NPVs) at the species level were consistently high, ranging from 98.10% to 100% (Table 5).

Table 6 compares the semi-quantitative results obtained from the PN panel (genomic abundance, copies/mL) with those from CC method (CFU/mL). PN panel results were stratified into two categories: low when the genomic abundance was 10^4^–10^5^ copies/mL (<10^6^) and high when it was ≥10^6^ copies/mL (10^6^–≥10^7^). Conventional culture results were similarly stratified, with bacterial growth of 10^4^ CFU/mL classified as low (<10^5^) and growth of ≥10^5^ CFU/mL (10^5^–10^6^) classified as high (≥10^5^). Cut-off values used for the stratification of molecular and CC results were defined according to both manufacturer recommendations for interpreting semi-quantitative results for bacteria [11] and a previously published manuscript by Mustafa Hellou and colleagues [22]. Comparison between genomic abundance and bacterial growth showed different patterns among bacteria, as described below.

For *S. aureus* and Enterobacterales, genomic abundance measured by the PN panel showed a strong correspondence with culture outcomes. Among *S. aureus*-positive samples, low genomic abundance was associated with absent culture growth in 58.97% of cases, whereas high genomic abundance aligned more closely with culture positivity, with 35.71% showing low growth and 50.00% showing high growth. A similar trend was observed for *Enterobacterales* (Table 6). Overall, these patterns indicate that higher genomic loads are strongly linked to increased bacterial viability.

Among non-fermenting Gram-negative bacteria, a strong association was observed between genomic abundance and culture results. Samples with low genomic levels showed no growth in 64.71% of cases, while 19.36% high-abundance detections corresponded to low growth and 74.19% to high growth in culture, highlighting a high level of concordance between molecular and CC methods.

For *Streptococcus* spp., *H. influenzae*, and *M. catarrhalis*, overall culture positivity was low, and only a moderate association was observed between genomic abundance and culture outcomes. Among *Streptococcus* spp., 92.3% of low-abundance samples and 57.9% of high-abundance samples showed no growth, with only 36.8% of high-abundance detections corresponding to strong culture growth. Similarly, *H. influenzae* and *M. catarrhalis* were rarely recovered by culture, with 87.0% and 56.5% of samples showing no growth at low and high genomic abundance, respectively; only 34.8% of high-abundance detections corresponded to strong culture growth.

Figure 2 shows the comparison of mean genomic abundance detected by the PN panel for +PN/+CC and +PN/−CC samples across all bacterial groups. In every group evaluated, +PN/+CC samples demonstrated significantly higher genomic abundance than +PN/−CC samples.

### 3.4. Resistance Genes

**Figure 3:**
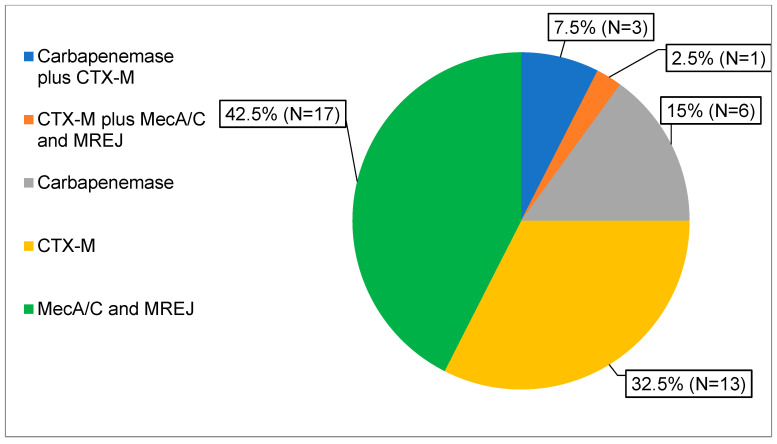
Distribution of resistance genes detected by PN panel. Detected genes were classified according to resistance type, as described in the legend.

A total of 44 resistance genes were detected by the PN panel in 40 of 410 respiratory specimens, with 4 samples showing simultaneous detection of two different resistance genes (Figure 3). The most frequently detected targets were *mecA/C* and MREJ (18 cases, 40.9%), followed by CTX-M (17 cases, 38.6%).

Among the 40 samples positive for resistance genes, 15 showed no bacterial growth by the CC method and were therefore excluded from further comparison. In the remaining 25 culture-positive isolates, the presence of resistance markers was confirmed in 20 samples using qualitative lateral-flow immunoassays for CTX-M and carbapenemases, and the LAMP technique for *mecA/C*.

Table 7 summarizes the concordance between the PN panel and the reference tests used for detecting resistance markers in culture isolates. Overall, for carbapenemases and *mecA/C* and MREJ, the PN panel demonstrated a positive predictive value (PPV) of 75% and a negative predictive value (NPV) of 100%. For CTX-M, concordance was higher, with a PPV of 90.91% and an NPV of 95.65%.

## 4. Discussion

In our study, we found that 54% of BAL samples from patients with suspected pneumonia were positive for at least one microbial target by PN panel, versus a positivity rate of CC method equal to 33.9%. Sensitivity, specificity, and accuracy of the PN panel demonstrated high diagnostic performance across the majority of bacterial targets. Specificity remained consistently elevated across all bacterial targets, while sensitivity values were more variable, reflecting heterogeneity in bacterial detection.

PN panel was already known to detect many more bacterial targets than the CC method [3,4,23]. These results were confirmed across different settings and sample types: Kosai et al. reported a threefold higher detection of bacterial and resistance targets in 57 BAL and mini-BAL specimens [3], Buchan et al. observed approximately a twofold increase in 259 BAL samples [4], and Lee et al. found a 20% higher detection of one or more bacterial targets in 59 endotracheal aspirates and BAL specimens [23].

Although the positivity rate of the PN panel in our study was significantly higher than that of the CC method, it did not exceed 55% overall. A possible explanation could be that the PN panel was performed on specimens collected from patients with suspected pneumonia, without distinguishing between those who were ultimately diagnosed with pneumonia and those who were not. This is supported by a recent publication of Mustafa Hellou and colleagues, which reported an increase in positivity rate of 20.2% (from 44.8% to 65%), focusing only on samples from patients ultimately diagnosed with pneumonia [22].

In our setting, *S. aureus* was the most frequently identified bacterium by both the PN panel and CC method, accounting for 27.39% (40/146). This finding is consistent with other studies from Europe and the United States, which reported a prevalence of *S. aureus* ranging from 20% to 28% [12,24]. In contrast, *H. influenzae* (73.8%, 31/42) and *S. pneumoniae* (64%, 16/25) exhibited the highest proportion of samples that were positive by the PN panel but negative by the CC method. These findings are consistent with those reported in previous studies [12,13]. On one hand, this low concordance between PN and CC could be explained by the fastidious nature of these bacteria; on the other hand, the under-detection by the CC method could be attributable to a prior antibiotic use before specimen collection, which could have affected bacterial growth [4,24]. Furthermore, asymptomatic carriage of these species within the respiratory tract should be taken into account, as it could partially explain molecular detection in the absence of culture-based growth. Five bacterial species (*Achromobacter xylosoxidans*, *Burkholderia cepacia* group, *Citrobacter koseri*, *Morganella morganii*, *S. maltophilia*) and one fungal species (*Aspergillus fumigatus*) were identified in 14 (3.4%) samples exclusively through the CC method, as these pathogens are not currently included among the PN panel targets. These findings are consistent with previous studies that reported the isolation of *S. maltophilia*, *A. xylosoxidans*, and *A. fumigatus* as etiological agents of pneumonia in immunocompromised patients and in intensive care unit (ICU) settings, including cases involving SARS-CoV-2 infection [25,26,27,28,29,30]. Consequently, it would be valuable to consider updating the PN panel by integrating these additional pathogens, which may improve its diagnostic coverage and accuracy for a wider range of respiratory infections. Moreover, this implementation could be particularly useful in hospital settings that serve as referral centers for patients with chronic lung diseases (such as cystic fibrosis, chronic obstructive pulmonary disease, asthma, and idiopathic pulmonary fibrosis), as in our real-life scenario. Accordingly, the appropriate use and interpretation of the PN panel for diagnosing LRTIs should be carefully guided by local epidemiology.

As reported by the manufacturer and previous studies, the genomic abundance (copies/mL) measured by the PN Panel is not equivalent to bacterial growth expressed in colony-forming units (CFU/mL) and does not always consistently correlate with the bacterial load measured by CC method [4,11,31]. Buchan and colleagues observed that in 77.2% of all cases of discordant quantification, the PN Panel values exceeded culture quantification by more than one logarithmic unit (>1 log) [4]. Similarly, Murphy and colleagues observed that PN Panel values exceeded culture quantification in 58.9% to 93.8% of specimens with bacterial growth below 10^6^ CFU/mL [31]. In our study, we observed higher genomic abundance in specimens that tested positive by both PN panel and CC method (concordant positive samples) compared to specimens that tested positive by PN panel but negative by CC method. Specifically, for non-fermenting Gram-negative bacteria, *S. aureus* and *Enterobacterales,* we observed that specimens with higher genomic abundance generally exhibit higher growth in culture, while for *Streptococcus* spp., *H. influenzae,* and *M. catarrhalis*, which are commonly considered members of normal respiratory microbiota, this kind of correlation was not observed. These results are consistent with those of Mustafa Hellou and colleagues, who suggested that a high genomic abundance accompanied by correspondingly high bacterial growth may serve as a marker of active infection or significant colonization, excluding components of normal respiratory microbiota [22].

Regarding resistance genes, only half of those detected by the PN panel (22/44) were confirmed by culture, with a prevalence of CTX-M–producing *Enterobacterales* and methicillin-resistant *S. aureus*. These findings are consistent with those reported by Gasli and colleagues, in which only 24 (57%) of the 42 genes detected by the PN panel were confirmed by routine antimicrobial susceptibility testing methods, with 71% of ESBL-producing *Enterobacterales* CTX-M and 29% of methicillin-resistant *S. aureus* [12]. Nevertheless, it is important to note that the absence of resistance genes detected by PN panel does not guarantee antibiotic susceptibility of the corresponding bacterial strains [5]. Therefore, culture and antimicrobial susceptibility testing may be required to ensure accurate antimicrobial treatment selection. This is particularly relevant for bacterial species exhibiting mutation-mediated resistance mechanisms, such as *S. pneumoniae* and *P. aeruginosa* [5,31]. Furthermore, resistance genes detected by the PN panel are not necessarily part of the genomic content of the detected pathogen, as they may originate from other colonizing bacteria rather than the pathogens responsible for the patient’s pneumonia [13].

The added value of our study lies in the large number of BAL samples collected during the study period, distinguishing it from other studies in the literature that have also included a broader range of respiratory sample types beyond BAL, including tracheal aspirates, expectorated, and induced sputum [4,15,22,23,32,33,34,35]. This approach enhances the reliability of our findings by focusing on a single, standardized sample type. Additionally, the exclusive use of BAL samples allows for a more homogeneous dataset, minimizing variability introduced by different sample types. This methodological rigor contributes to the robustness and validity of our study’s conclusions.

This study also has some limitations. First, we could not collect clinical data, such as symptoms and clinical outcomes. Consequently, we were unable to distinguish between patients ultimately diagnosed with pneumonia (based on microbiological and imaging confirmation) and those without, limiting a comprehensive evaluation of the PN panel’s impact on patient management. Second, the lack of specimen quality assessment and data on prior antibiotic therapy, both of which can affect bacterial growth, limited our ability to explain discrepancies between the PN panel and CC method. Third, PN Panel results for atypical bacteria and viruses were not compared using other molecular assays. When clinicians suspect atypical pneumonia or viral pneumonia, targeted multiplex assays different from PN panel are performed as part of our diagnostic workup.

## 5. Conclusions

Our study confirms that the PN panel provides enhanced detection of bacterial pathogens in BAL samples from patients with suspected pneumonia, demonstrating a 20% higher positivity rate compared to CC. The PN panel showed high specificity across all bacterial targets and generally strong sensitivity, with semi-quantitative results offering useful guidance to distinguish true pathogens from commensal microbiota.

Certain pathogens not yet included in the PN panel (e.g., *S. maltophilia*, *A. fumigatus*) were detected by culture in the 3.4% of samples, suggesting the need to consider expanding panel targets for comprehensive respiratory pathogen coverage.

Detection of resistance genes by the PN panel showed moderate concordance with culture-based confirmation, even though culture and susceptibility testing remain essential for guiding targeted therapy.

Overall, the PN panel represents a valuable adjunct to conventional diagnostics for LRTIs. Its rapid, semi-quantitative detection enhances pathogen identification, thereby improving antimicrobial stewardship and enabling more targeted and appropriate antibiotic therapy. Interpretation of PN panel results should be performed in the context of specimen quality, local epidemiology, and in combination with conventional culture methods, especially when assessing bacterial viability and resistance.

## Figures and Tables

**Table 1:** Patient characteristics and distribution of respiratory specimens across care units.

Characteristics and Critical Care Units	Overall	2022	2023	2024	*p* -Value
Patients (N)	343	94	110	139	
Males (N, %)	238 (69.4)	67 (71.3)	80 (72.7)	91 (65.5)	0.42 *
Age (year, median)	60 (48–68)	63 (52–69)	57 (46–68)	60 (46–68)	0.25 **
Specimens (N)	410	115	133	162	
Hematology (N, %)	31 (7.6)	13 (11.3)	7 (5.3)	11 (6.8)	0.06 *
Intensive care (N, %)	245 (59.8)	75 (65.2)	81 (60.9)	89 (54.9)	
Medicine (N, %)	115 (28.0)	26 (22.6)	36 (27.1)	53 (32.7)	
Surgery (N, %)	19 (4.6)	1 (0.9)	9 (6.7)	9 (5.6)	

* The *p*-value was calculated using the Chi-square test. ** The *p*-value was calculated using the Kruskal-Wallis test.

**Table 2:** Prevalence of detected pathogens using PN panel tests.

Targets	PN Panel N (%)
**Bacteria**	
*Acinetobacter calcoaceticus-baumannii* complex	9 (2.20)
*Enterobacter cloacae* complex	10 (2.44)
*Escherichia coli*	28 (6.83)
*Haemophilus influenzae*	42 (10.24)
*Klebsiella aerogenes*	4 (0.98)
*Klebsiella oxytoca*	5 (1.22)
*Klebsiella pneumoniae* group	20 (4.88)
*Moraxella catarrhalis*	4 (0.98)
*Proteus* spp.	6 (1.46)
*Pseudomonas aeruginosa*	39 (9.51)
*Serratia marcescens*	8 (1.95)
*Staphylococcus aureus*	67 (16.34)
*Streptococcus agalactiae*	2 (0.49)
*Streptococcus pneumoniae*	25 (6.10)
*Streptococcus pyogenes*	5 (1.22)
**Atypical bacteria**	
*Chlamydia pneumoniae*	1 (0.24)
*Legionella pneumophila*	5 (1.22)
*Mycoplasma pneumoniae*	4 (0.98)
**Viruses**	
*Adenovirus*	4 (0.98)
*Coronavirus (229E, OC43, HKU1, NL63)*	12 (2.93)
*Influenza Virus A*	21 (5.12)
*Influenza Virus B*	0 (0.00)
*MERS-CoV*	0 (0.00)
*Metapneumovirus*	8 (1.95)
*Parainfluenza Virus*	5 (1.22)
*Respiratory Syncytial Virus (RSV)*	4 (0.98)
**Total samples**	221 (54)

**Table 3:** Prevalence of detected pathogens using CC method.

	Culture N (%)
**Pathogens included in PN panel**	
*Acinetobacter calcoaceticus-baumannii* complex	8 (1.95)
*Enterobacter cloacae* complex	7 (1.71)
*Escherichia coli*	19 (4.63)
*Haemophilus influenzae*	11 (2.68)
*Klebsiella aerogenes*	2 (0.49)
*Klebsiella oxytoca*	2 (0.49)
*Klebsiella pneumoniae* group	20 (4.88)
*Moraxella catarrhalis*	2 (0.49)
*Proteus* spp.	5 (1.22)
*Pseudomonas aeruginosa*	35 (8.54)
*Serratia marcescens*	4 (0.98)
*Staphylococcus aureus*	40 (9.76)
*Streptococcus agalactiae*	0 (0.00)
*Streptococcus pneumoniae*	9 (2.20)
*Streptococcus pyogenes*	0 (0.00)
**Pathogens not included in PN panel**	
*Aspergillus fumigatus*	1 (0.24)
*Achromobacter xylosoxidans*	1 (0.24)
*Burkholderia cepacia* group	1 (0.24)
*Citrobacter koseri*	3 (0.73)
*Morganella morganii*	1 (0.24)
*Stenotrophomonas maltophilia*	8 (1.95)
**Total samples**	138 (33.9)

**Table 4:** Sensibility, specificity, and accuracy of PN panel compared with CC method.

Bacteria	Sensibility (%, CI)	Specificity (%, CI)	Accuracy (%, CI)
*Acinetobacter calcoaceticus-baumannii* complex	87.5 (47.35–99.68)	99.5 (98.21–99.94)	99.27 (97.88–99.85)
*Enterobacter cloacae* complex	57.14 (18.41–90.10)	98.51 (96.79–99.45)	97.8 (95.87–98.99)
*Escherichia coli*	89.47 (66.86–98.70)	97.19 (95.02–98.59)	96.83 (94.64–98.30)
*Haemophilus influenzae*	100 (71.51–100.00)	92.23 (89.15–94.66)	92.44 (89.44–94.81)
*Klebsiella aerogenes*	100 (15.81–100.00)	99.51 (98.24–99.94)	99.51 (98.25–99.94)
*Klebsiella oxytoca*	100 (15.81–100.00)	99.26 (97.87–99.85)	99.27 (97.88–99.85)
*Klebsiella pneumoniae* group	75 (50.90–91.34)	98.72 (97.03–99.58)	97.56 (95.56–98.82)
*Moraxella catarrhalis*	100 (15.81–100.00)	99.51 (98.24–99.94)	99.51 (98.25–99.94)
*Proteus* spp.	100 (47.82–100.00)	99.75 (98.63–99.99)	99.76 (98.65–99.99)
*Pseudomonas aeruginosa*	80 (63.06–91.56)	97.07 (94.81–98.53)	95.61 (93.15–97.38)
*Serratia marcescens*	100 (39.76–100.00)	99.01 (97.50–99.73)	99.02 (97.52–99.73)
*Staphylococcus aureus*	100 (91.19–100.00)	92.7 (89.56–95.14)	93.41 (90.56–95.62)
*Streptococcus agalactiae*	NA *	99.51 (98.25–99.94)	NA *
*Streptococcus pneumoniae*	100 (66.37–100.00)	96.01 (93.60–97.70)	96.1 (93.74–97.75)
*Streptococcus pyogenes*	NA *	98.78 (97.18–99.60)	NA *
**Total**	89.02 (83.21–93.36)	97.86 (97.46–98.21)	97.63 (97.21–97.99)

* NA = not applicable.

**Table 5:** Positive predictive value and negative predictive value of PN panel compared with CC method.

Bacteria	+PN/+CC	−PN/−CC	+PN/−CC	−PN/+CC	PPV * (%, CI)	NPV * (%, CI)
*Acinetobacter calcoaceticus-baumannii* complex	7	400	2	1	77.8 (46.15–93.46)	99.8 (98.46–99.96)
*Enterobacter cloacae* complex	4	397	6	3	40 (19.37–64.92)	99.3 (98.25–99.68)
*Escherichia coli*	17	380	11	2	60.7 (45.83–73.85)	99.5 (98.08–99.96)
*Haemophilus influenzae*	11	368	31	0	26.2 (20.19–33.23)	100 (99.00–100)
*Klebsiella aerogenes*	2	406	2	0	50 (20.06–79.94)	100 (99.10–100)
*Klebsiella oxytoca*	2	405	3	0	40 (17.76–67.30)	100 (99.09–100)
*Klebsiella pneumoniae* group	15	385	5	5	75 (54.78–88.14)	98.7 (97.30–99.40)
*Moraxella catarrhalis*	2	406	2	0	50 (20.06–79.94)	100 (99.10–100)
*Proteus* spp.	5	404	1	0	83.3 (41.38–97.25)	100 (99.09–100)
*Pseudomonas aeruginosa*	28	364	11	7	71.8 (58.15–82.34)	98.1 (96.40–99.02)
*Serratia marcescens*	4	402	4	0	50 (27.39–72.61)	100 (99.09–100)
*Staphylococcus aureus*	40	343	27	0	59.7 (50.75–68.05)	100 (98.93–100)
*Streptococcus agalactiae*	0	408	2	0	NA **	100 (99.10–100)
*Streptococcus pneumoniae*	9	385	16	0	36 (25.81–47.63)	100 (99.09–100)
*Streptococcus pyogenes*	0	405	5	0	NA **	98.78 (97.18–99.60)
**Total**	146	5858	128	18	89.02 (83.21–93.36)	97.86 (97.46–98.21)

* PPV = Positive Predictive Value. NPV = Negative Predictive Value. CI = Confidence Interval. ** NA = not applicable.

**Table 6:** Correlation rate between genomic abundance detected by PN panel and CC method.

	Genomic Abundance (Low [<10^6^]) (High [≥10^6^])	No Growth in Culture (N, %)	Low Growth in Culture ([<10^5^]) (N, %)	High Growth in Culture ([≥10^5^]) (N, %)
*Staphylococcus aureus*	Low (*N* = 39)	23 (58.97)	14 (35.90)	2 (5.13)
	High (*N* =28)	4 (14.29)	10 (35.71)	14 (50.00)
*Streptococcus* spp. *	Low (*N* = 13)	12 (92.31)	0 (0.0)	1 (7.69)
	High (*N* = 19)	11 (57.89)	1 (5.26)	7 (36.85)
*Enterobacterales*	Low (*N* = 46)	25 (54.37)	17 (36.96)	4 (8.69)
	High (*N* = 35)	7 (20.00)	8 (22.86)	20 (57.14)
Non-Fermenting Gram-Negative Bacteria **	Low (*N* = 17)	11 (64.71)	6 (35.29)	0 (0.0)
	High (*N* = 31)	2 (6.45)	6 (19.36)	23 (74.19)
*Haemophilus influenzae* and *Moraxella catarrhalis*	Low (*N* = 23)	20 (86.95)	2 (8.70)	1 (4.35)
	High (*N* = 23)	13 (56.52)	2 (8.70)	8 (34.78)

* *Streptococcus* spp. = *S. pyogenes*, *S. agalactiae*, *S. pneumoniae*. *Enterobacterales* = *Enterobacter cloacae* complex, *Escherichia coli*, *Klebsiella aerogenes*, *Klebsiella oxytoca*, *Klebsiella pneumoniae* group, *Proteus* spp., *Serratia marcescens*. ** Non-Fermenting Gram-Negative Bacteria = *Pseudomonas aeruginosa* and *Acinetobacter calcoaceticus-baumannii* complex.

**Table 7:** Concordance rate between PN panel and culture-based tests for resistance markers.

	+PN/−CC	+PN/−CC	−PN/+CC	−PN/−CC	PPV * (%, CI)	NPV * (%, CI)
Carbapenemase	3	1	0	38	75 (30.24–95.41)	100.00 (90.75–100.00)
CTX-M	10	1	1	22	90.91 (59.30–98.56)	95.65 (77.21–99.30)
mecA/C and MREJ	9	3	0	28	75 (50.58–89.79)	100.00 (87.66–100.00)

* PPV = Positive Predictive Value. NPV = Negative Predictive Value. CI = Confidence Interval.

## Data Availability

The original contributions presented in this study are included in the article. Further inquiries can be directed to the corresponding author.

## Abbreviations

The following abbreviations are used in this manuscript:

LRTIs	Low Respiratory Tract Infections
PN	BIOFIRE FilmArray^®^ Pneumonia
CC	Conventional Culture
AST	Antimicrobial susceptibility Test
BAL	Bronchoalveolar Lavage
LAMP	Loop-mediated isothermal Amplification
PPV	Positive Predictive Value
NPV	Negative Predictive Value
CI	Confidence Interval
NA	Not Applicable
